# Longitudinal Analysis of Vulvovaginal Bacteriome Following Use of Water- and Silicone-Based Personal Lubricants: Stability, Spatial Specificity, and Clinical Implications

**DOI:** 10.3390/microorganisms14010082

**Published:** 2025-12-30

**Authors:** Jose A. Freixas-Coutin, Jin Seo, Lingyao Su, Sarah Hood

**Affiliations:** 1 Reckitt Health US LLC, 1 Philips Pkwy, Montvale, NJ 07645, USA; jin.seo@reckitt.com; 2 RB (Shanghai) Technology Co., Ltd., Shanghai 200030, China; lynn.su@reckitt.com; 3 Reckitt Benckiser Healthcare Ltd., Hull HU8 7DS, UK; sarah.hood@reckitt.com

**Keywords:** vulvovaginal bacteriome, personal lubricants, menopause, 16S rRNA gene, vaginal dryness

## Abstract

The vulvovaginal microbiome is a complex and dynamic ecosystem of microorganisms. The potential effects of common personal lubricants on its balance, which have implications for reproductive health, are still unknown. This study longitudinally assessed the impact of two commercially available lubricants on the composition and stability of the vaginal and vulvar bacteriome. Paired vaginal and vulvar swabs were collected at baseline and after repeated lubricant use, and the bacteriome was assessed using 16S rRNA gene amplicon sequencing. Alpha and beta diversity were assessed using Shannon entropy and Bray–Curtis dissimilarity, respectively. The results showed that the vaginal bacteriome was dominated by *Lactobacillus* and *Firmicutes*, while vulvar communities were more diverse and had higher abundances of *Prevotella*, *Finegoldia*, and *Peptoniphilus*. Both alpha and beta diversity measures indicated that the vaginal and vulvar bacteriome remained largely stable even after repeated lubricant use. Minor and non-significant changes in genus-level composition were observed, particularly in the vulvar samples. A moderate but significant correlation (Mantel r = 0.274, *p* = 0.001) was also observed between the vaginal and vulvar bacteriome. Overall, this study shows that short-term, repeated use of the water-based lubricant and the silicone-based lubricant tested in this study does not significantly disrupt the vaginal or vulvar bacteriome.

## 1. Introduction

The human vulvovaginal microbiome is a collection of living microorganisms, including bacteria, fungi, and viruses, that naturally inhabit the human vulva skin and vagina. In a healthy vulvovaginal environment, these microorganisms work in harmony with the body and play a vital role in maintaining pH balance, preventing the growth of pathogens, and strengthening the immune system [1,2]. The most important and dominant bacterial group in the vaginal bacteriome is *Lactobacillus*, which produces lactic acid to keep the vaginal environment acidic. This acidic state is vital for preventing the growth of harmful bacteria, fungi, and viruses [3]. When the balance of the microbiome is disrupted, due to factors such as menopause, personal care product use, antibiotics, hormonal changes, stress, or an unhealthy diet, it can create a favorable environment for pathogenic microorganisms, potentially leading to vaginal infections or even reproductive disorders [4]. The balance of the vaginal microbiome is not only effective in maintaining women’s daily health but also plays a pivotal role in fertility and pregnancy. Studies have shown that women of reproductive age with a healthy vaginal microbiome dominated by *Lactobacillus* have a greater chance of successful fertilization, embryo implantation, and a healthy pregnancy [5,6]. In addition to general reproductive health and pregnancy outcomes, the vaginal microbiome also influences specific physiological processes that affect fertility and help prevent infections [7]. The protective functions of lactobacilli extend beyond maintaining acidity; they also create an environment that supports sperm survival and reduces the risk of infection [5]. These roles suggest that a healthy microbiome holds broader and more prominent importance throughout different stages of a woman’s life, from fertility to postmenopausal health [1]. By producing lactic acid, lactobacilli suppress the growth of pathogenic microorganisms and help maintain conditions that favor reproductive health. During intercourse, the slightly alkaline nature of semen temporarily neutralizes vaginal acidity, allowing sperm to survive and move toward the uterus. [8]. A healthy vaginal microbiome also helps prevent complications during pregnancy, including preterm labor, premature rupture of membranes, and ascending uterine infections [9]. Moreover, some studies have explored the association between vaginal microbiome imbalances and recurrent miscarriage [10,11], though further research is needed to clarify this relationship.

Typically, vaginal dryness is caused by decreased estrogen levels due to menopause, but it can also result from other factors such as childbirth, breastfeeding, cancer treatments, certain medications, autoimmune disorders, and lifestyle habits such as smoking or douching [12,13]. Vaginal dryness can cause discomfort and pain during sex. For this reason, a person may use vaginal gels and lubricants [14]. One treatment is to use over-the-counter personal lubricants or moisturizers inside the vagina 2–3 times a week to restore moisture. In general, there are several types of lubricants: water-based lubricants, silicone-based lubricants, oil-based lubricants, and natural lubricants [14]. Previous studies investigating the effects of personal lubricants on the vaginal microbiome have suggested that repeated use of water-based lubricants over several weeks does not substantially alter microbial diversity or relative abundance, although some species-level changes may occur depending on baseline microbiota composition [15,16,17,18,19,20]. These findings underscore that the vaginal microbiome is largely resilient to short-term lubricant use, while factors such as age, baseline microbial composition, and lubricant formulation may influence subtle microbial fluctuations.

In the present study, a longitudinal analysis of the bacteriome was conducted on paired vaginal and vulvar swab samples from participants who used two types of personal lubricants, a water-based lubricant (Tingling) and a silicone-based lubricant (Warming Liquid, herein referred to as Warming), using 16S rRNA gene amplicon sequencing. The study was designed to determine whether short-term use of two personal lubricants independently alters the vaginal or vulvar microbiome. To this end, the study aimed to characterize the relative taxonomic composition and diversity of the bacteriome at both vaginal and vulvar (i.e., labia) sites; assess intra-individual and temporal variation in community structure; identify taxa contributing to the similarity between sites; and examine associations between bacteriome composition and host clinical parameters, including pH, BMI, and age. Given differences in base formulation, chemesthetic ingredients, and preservative systems, lubricants could potentially influence low-abundance microbial taxa or microbial interactions by transiently modifying the local mucosal environment, while core *Lactobacillus*-dominated communities were expected to remain largely stable. In addition, a detailed overview of the vulvovaginal bacteriome and its stability over time was provided through comprehensive bioinformatic and statistical analyses, including dada2-based raw data processing, linear mixed-effects (LME) modeling, PERMANOVA, and zero-inflated Generalized LME modeling.

## 2. Materials and Methods

### 2.1. Clinical Study Design

This open-label, two-arm, parallel-design clinical trial was conducted from February 2023 to May 2023 by SGS Proderm GmbH in Hamburg, Germany. The study was performed in accordance with the Clinical Investigation Plan (CIP), the Declaration of Helsinki, International Council for Harmonization standards, Good Clinical Practice, ISO 14155:2020, and applicable regulatory standards. The trial was registered at ClinicalTrials.gov (Identifier: NCT05644444). Written informed consent was obtained from all female participants and their male partners prior to enrollment. The study complied with the European Union General Data Protection Regulation.

The study design, including participant inclusion and exclusion criteria, was the same as that described in previously published research on the clinical study outcomes. [21]. A total of 66 female participants, aged 18–65 years, were enrolled and randomized in a 1:1 ratio to receive one of two commercially available personal lubricants. Randomization was performed using permuted blocks of fixed size (*n* = 4) and stratified by menopausal status (premenopausal vs. postmenopausal). The study was designed to achieve 90% statistical power, with 33 participants per group to ensure at least 30 per group after accounting for dropouts, targeting a meaningful improvement in sexual function (FSFI total score increase of ≥4 points from baseline), which was the prespecified primary endpoint published elsewhere [21].

The lubricants were suitable for vaginal, oral, and anal use and met the World Health Organization’s interim guidelines for personal lubricants, including osmolality ≤1200 mOsm/kg and an acidic pH within the healthy vaginal range (3.8–4.5) [21]. Two distinct lubricant formulations were evaluated in this study: a water-based “Tingling” lubricant and a silicone-based “Warming” lubricant. Participants were instructed to use approximately 3 g of lubricant per application, limited to four applications per day, and mandated to use it at least weekly for four weeks during sexual intercourse. Premenopausal participants who menstruated during the 4-week treatment phase were allowed to temporarily pause the use of assigned lubricant during intercourse; such interruptions were documented in their participant diaries without compromising their required minimum weekly sexual activity, which could be met by engaging in intercourse twice within a single week to accommodate menstrual breaks.

Beyond the difference in base formulation, the two lubricants also differ in their sensate (chemesthetic) ingredients and overall composition. The water-based lubricant contains a cyclic carboxamide (<1%), whereas the silicone-based lubricant contains vanillyl butyl ether (<1%). These ingredients activate different transient receptor potential (TRP) ion channels, which mediate sensations ranging from cooling to warmth [22,23]. The lubricants also contained different preservative systems suited to their respective formulations; however, these differences appeared to have minimal impact on the vaginal and vulvar microbiome, as no significant compositional changes were observed during the study.

Key inclusion criteria included being in a monogamous relationship with the opposite sex, using effective contraception (for premenopausal participants), and reporting mild to moderate vaginal dryness and/or dyspareunia. For postmenopausal participants, eligibility was the absence of menstrual periods for at least 12 months due to natural or medical reasons. Exclusion criteria included current pregnancy or breastfeeding, intention to become pregnant, known allergies to lubricants, and any active vaginal or urinary tract infection. Those taking medications that interfered with vaginal moisture were also excluded. Detailed inclusion and exclusion criteria for the participants are described in the previous publication [21].

As part of the screening process, participants rated the severity of vaginal dryness and dyspareunia using a 4-point verbal rating scale (0 = no dryness/pain, 3 = severe dryness/pain). Eligible participants completed two phases of the study: an initial tolerance phase involving a subset of 22 participants, followed by a treatment phase that included all eligible participants. During the tolerance phase, participants were monitored for side effects and product compliance. The treatment phase assessed clinical endpoints including product perception and improved intimacy which were published previously [21].

### 2.2. Sample Collection, DNA Extraction and Sequencing

Participants were assigned to one of two personal lubricants: Tingling (n = 33) or Warming (n = 33). A total of 262 swab samples were collected from 66 female participants by a qualified clinician at baseline (after a 7-day washout without vaginal penetration or external products and before any lubricant use) and again at the final visit after 4 weeks of lubricant use. Vaginal swabs were collected from the vaginal walls 3–5 cm from the external orifice toward the lateral fornix, and vulvar swabs from the labia majora (hereafter referred to as the vulva), with all swabs rotated for full coverage. One participant in the Warming group did not complete the study; therefore, vaginal and vulvar swabs were not collected for this participant at the final time point. In total, 131 samples were collected from the vaginal area and 131 from the vulvar area. DNA was extracted from all swabs using standard methods available at Clinical Microbiomics (Copenhagen, Denmark). The purified DNA was used for amplicon sequencing targeting the highly variable V1–V3 regions of the 16S rRNA gene to analyze bacterial community composition. The V1–V3 region was chosen due to its higher resolution and ability to detect a wider range of species compared to the V3-V4 region used in previous studies [24,25].

Amplicon libraries were generated using the NEXTFLEX^®^ 16S V1–V3 Amplicon-Seq Kit (Bioo Scientific, Austin, TX, USA) according to the manufacturer’s protocol. The primers targeting the V1–V3 region of the 16S rRNA gene were as follows: forward primer 5′-CTCTTTCCCTACACGACGCTCTTCCGATCTAGAGTTTGATCCTGGCTCAG-3′ and reverse primer 5′-CTGGAGTTCAGACGTGTGCTCTTCCGATCTGTATTACCGCGGCTGCTGG-3′. The initial PCR amplification protocol began with a denaturation step at 98 °C for 4 min, followed by 8 cycles of 30 s at 98 °C (denaturation), 30 s at 60 °C (annealing), and 30 s at 72 °C (extension), and finally terminated with a final extension at 72 °C for 4 min. After purification, amplicons were indexed with Illumina-compatible barcodes using a similar thermal cycling profile but increased to 20 cycles. The resulting libraries were then quantified and quality-assessed using fluorescence-based quantification and electrophoresis methods. After validation, the libraries were normalized, denatured, and pooled for sequencing using Illumina V3 chemistry according to the company’s standard protocols (Illumina, San Diego, CA, USA). Sequencing was performed on the Illumina MiSeq platform using paired end reads (2 × 301 cycles). Sequencing was completed in July, 2023 under the ISO/IEC 17025:2017 license of Clinical Microbiomics for microbiological testing (valid from 8 February 2021 to 28 February 2025).

### 2.3. Microbiome Analysis

All bioinformatics and statistical analyses were performed in RStudio (version 2025.5.0.496). The raw paired-end 16S rRNA gene sequences (V1–V3 region) were handled via dada2 package (version 1.36.0) to deduce amplicon sequence variants (ASVs). Non-biological reads corresponding to Illumina markers and 16S amplification primers were removed from the raw reads via *cutadapt* function. Reads with a Phred quality score of less than 10 and a length of less than 100 bp were filtered using the *filterAndTrim* function of dada2. Since the length of the V1–V3 amplicon exceeded the maximum paired-end sequencing read length of the MiSeq instrument (301 × 2 bp), the forward and reverse reads could not be merged. Therefore, only the forward reads were employed for downstream analyses. Forward data were further de-noised, checked for sequencing artifacts and filtered for chimeras using the *removeBimeraDenovo* function in dada2, which also converted them to ASV. Taxonomic classification of the ASV was accomplished via the *assignTaxonomy* function on the SILVA high-quality rRNA gene database (version 138) with a minimum bootstrap confidence of 50%. Species-level classifications were adjusted with the *addSpecies* function and required 100% bootstrap support to correct for potential misclassification due to the resolution limitations of 16S rRNA gene. ASVs that were not classified at the phylum or genus level, as well as those classified as mitochondrial, were dismissed from further analysis.

The resulting ASV count table was merged with taxonomic assignments and clinical metadata using the *phyloseq* package (version 1.52.0). Taxa that were present at least three times or less in 20% of the samples were removed to minimize noise from low-abundance taxa. Alpha diversity was determined using Shannon entropy using the vegan package (version 2.7.1), tested for significance via Wilcoxon paired tests and visualized across time and treatment groups. Beta diversity was assessed based on Bray–Curtis dissimilarity, visualized using principal coordinate analysis (PCoA), and tested for significant differences using PERMANOVA (*adonis2*, vegan) with participant as a random effect to account for repeated measurements. Homogeneity of distributions was measured via PERMDISP to ensure that differences were due to community diversity and not dispersion. *p* values from diversity and composition analyses were adjusted using the false discovery rate (FDR) method.

Since there are limitations in species-level resolution in the 16S V1–V3 region, differential abundance analyses were conducted at the genus level. Genus-level counts were transformed to centered log ratio (CLR) using the microbiome package (version 1.30.0). Generalized LME models (via glmmTMB package, version 1.1.12) evaluated the impact of time point (baseline vs. final) on CLR-transformed abundance values, including participant as a random effect. Pairwise comparisons and estimated marginal means were determined with *emmeans* and Benjamini–Hochberg corrections were also operated. To examine differences between anatomical sites (vaginal vs. vulvar), Mantel tests were operated on CLR Euclidean distance matrices to evaluate overall similarity, along with Spearman correlations for genus-specific associations. Differential enrichment analyses between anatomical sites were conducted via the *run_lefse* function of the microbiomeMarker package (version 1.8.0), which implements linear discriminant effect size (LEfSe) analysis.

Finally, correlations between bacteriome composition and clinical parameters (pH, BMI, age) at the genus level were modeled using generalized LME with zero inflation (glmmTMB package, version 1.1.12). Model validation, including dispersion, zero inflation, and outlier detection, was conducted via the DHARMa package (version 0.4.7). Effect estimates at the genus level were obtained using *broom.mixed* function, and Bonferroni correction was operated for multiple testing. Correlation results are reported separately by clinical variable and swab location in Supplementary Tables S5–S10 as these were non-significant (*p* > 0.05).

## 3. Results

**Figure 1:**
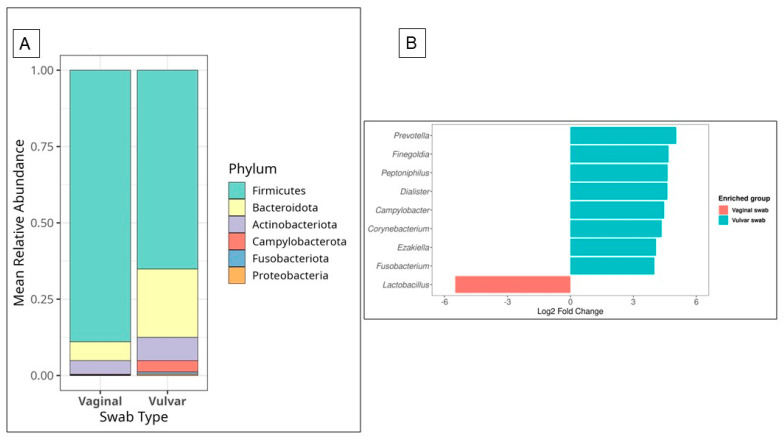
Composition and differential abundance of the vaginal and vulvar bacteriome at baseline. (**A**) The vaginal bacteriome was predominantly composed of *Firmicutes*, whereas the vulvar bacteriome exhibited greater diversity, with notable contributions from *Bacteroidetes*, *Actinobacteria*, and other phyla in addition to Firmicutes. (**B**) Differential abundance analysis (*p* < 0.05, |log_2_ fold change| > threshold) at the genus level revealed *Lactobacillus* enrichment in vaginal samples, while *Prevotella*, *Finegoldia*, and *Peptoniphilus* were significantly enriched in vulvar samples.

The overall composition of the vaginal and vulvar bacteriome differed in their microbial profiles. At the phylum level, the vaginal bacteriome was predominantly composed of Firmicutes, while the vulvar bacteriome was more diverse, with Firmicutes, Bacteroidetes, Actinobacteria, and Proteobacteria. These observations are based on relative abundance and are intended for descriptive purposes only (Figure 1A). At the genus level, differential abundance analysis using LEfSe showed that vaginal samples were rich in *Lactobacillus*, while vulvar samples were rich in *Prevotella*, *Finegoldia*, *Peptoniphilus*, *Dialister*, *Campylobacter*, *Corynebacterium*, *Escherichia*, and *Fusobacterium* (Supplementary Table S11 and Figure 1B).

**Figure 2:**
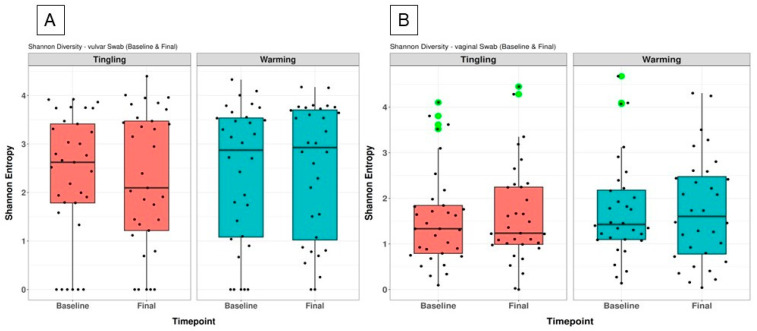
Shannon alpha diversity of vulvar and vaginal bacteriome at baseline and final timepoints, stratified by lubricant type. (**A**) Vulvar and (**B**) vaginal samples were analyzed for Shannon diversity across Tingling and Warming lubricant groups. No significant differences were observed between timepoints within each lubricant group. Outliers are indicated in green.

Figure 2 shows the alpha diversity measures conducted via Shannon entropy, which reveals anatomical and treatment-related trends. Vulvar samples consistently showed high and stable Shannon variability in both the Tingling and Warming treatment groups, although this difference was not significant between baseline and final time points (Figure 2A). In contrast, vaginal samples had lower baseline variability but showed a modest increase after the intervention, especially in the Tingling group, although this change was also not significant between baseline and final time points (Figure 2B).

Overall, alpha diversity remained stable over time in both anatomical sites and lubricant conditions as revealed by Wilcoxon test (Supplementary Table S1). Vulvar bacteriome exhibited higher alpha diversity compared with vaginal bacteriome.

**Figure 3:**
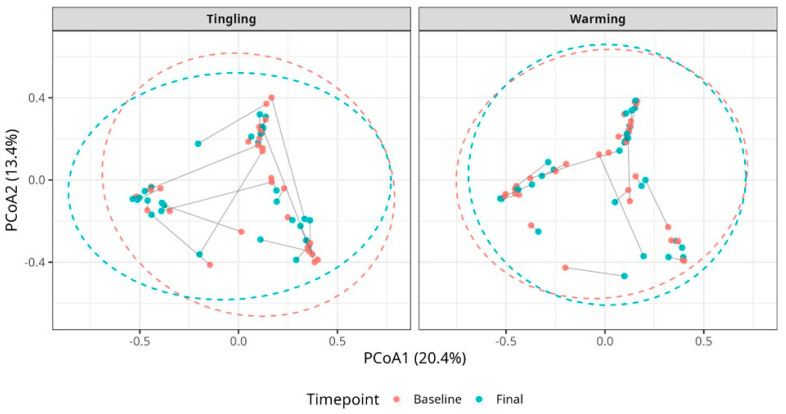
Principal coordinate analysis (PCoA) plots of vaginal samples at baseline and final timepoints for the Tingling and Warming lubricant groups. Each point represents an individual sample and connecting lines represent paired samples from the same individual. Dashed ellipses represent 95% confidence regions. No significant clustering was observed, indicating minimal changes in community composition over time.

**Figure 4:**
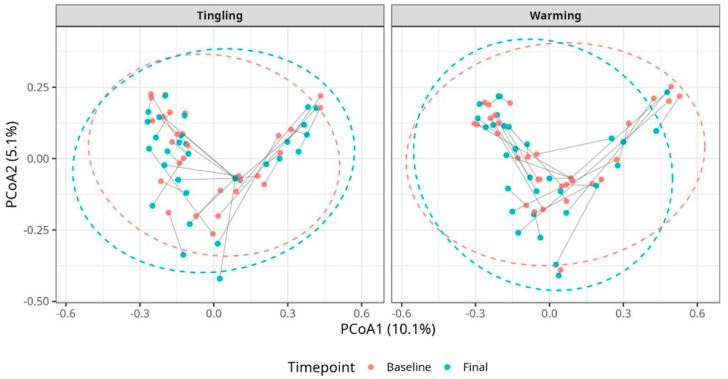
Principal coordinate analysis (PCoA) plots of vulvar samples at baseline and final time points from participants grouped in Tingling and Warming lubricant. Each dot represents a sample, colored by time point (red = baseline, blue = final). Lines connect paired samples from a participant. Dashed ellipses represent 95% confidence regions. No significant clustering was observed, indicating minimal changes in community composition over time.

The results from beta diversity analysis using Bray–Curtis intervals and principal coordinate analysis (PCoA) showed no significant clustering of samples by time point for either treatment group in vaginal or vulvar samples (Figure 3 and Figure 4). As shown in Figure 3, the plots from vaginal samples show a high degree of overlap between baseline and final samples, indicating no significant changes in overall microbial population diversity after lubricant use.

The results from the PERMANOVA tests were also non-significant (FDR-adjusted *p* > 0.05), and PERMDISP did not confirm any differences in dispersion (Supplementary Table S2). Similarly, the results from the vulvar samples confirmed that beta diversity was stable over time, with clustering at the individual level, but there was no clear separation between time points upon the treatments (Figure 4).

To account for the compositional nature of the data, statistical testing was performed using linear mixed-effects (LME) models on centered log-ratio (CLR)-transformed genus-level abundances between baseline and final time points for each lubricant group (Tingling and Warming) and anatomical site (vagina and vulva). These models did not detect any statistically significant changes in microbial composition over time (Supplementary Tables S3 and S4), indicating that product use had minimal impact on the bacteriome within the study period.

**Figure 5:**
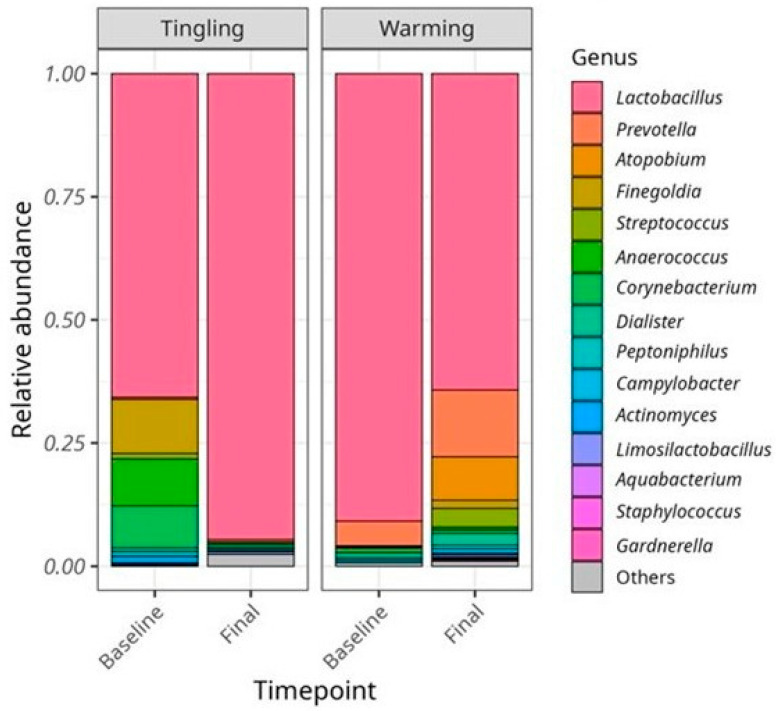
Relative abundance of the top 15 bacterial taxa at the genus level in vaginal samples at baseline and at the end of the study from participants who used the Tingling and Warming lubricants. Each bar represents the mean relative abundance of bacterial taxa across participants. *Lactobacillus* was dominant in all vaginal samples over time, indicating high stability. Only minor and non-significant fluctuations in the less abundant genera were observed after lubricant use.

As shown in the bar graphs of relative abundance at the genus level (Figure 5), *Lactobacillus* remained dominant in vaginal samples at both baseline and final time points. A slight visual increase in some anaerobic genera, such as *Prevotella*, *Atopobium*, and *Finegoldia*, was observed in the Warming group. However, it is important to note that these stacked bar graphs reflect relative abundances within each sample, meaning that apparent increases in a taxon may reflect a true increase, a decrease in other taxa, or both. Moreover, baseline differences between the lubricant groups make it difficult to attribute any observed differences between groups solely to lubricant composition. Therefore, these visualizations are intended for illustrative purposes only and should not be interpreted as evidence of inter-group compositional changes.

**Figure 6:**
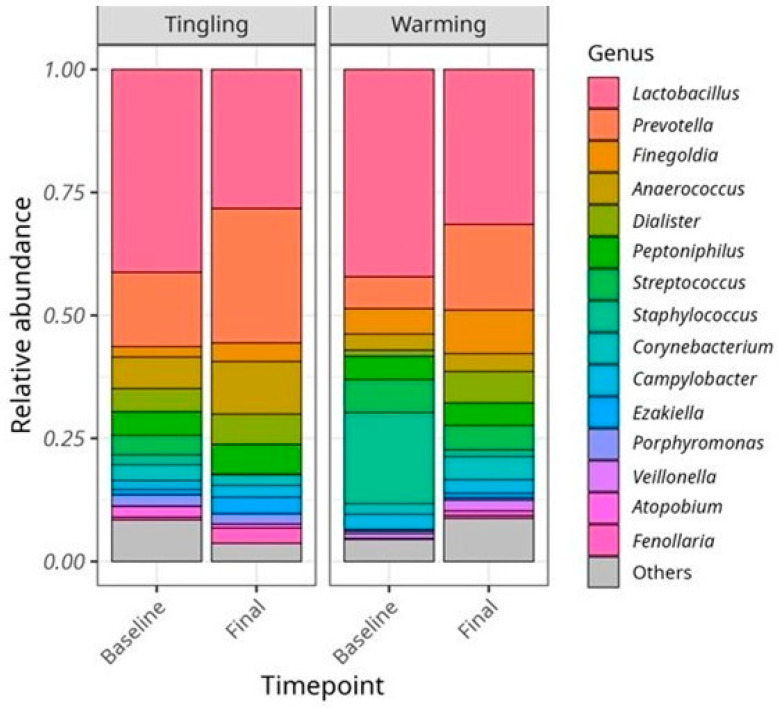
Relative abundance of the top 15 bacterial taxa at the genus level in vulvar samples at baseline and at the end of the study from participants who used Tingling and Warming lubricants. In contrast to the vaginal bacteriome, the vulvar bacteriome showed higher diversity and was more abundant in anaerobic genera such as *Prevotella*, *Finegoldia*, and *Peptoniphilus*. Changes between baseline and endpoint were minimal and non-significant.

Similarly, in vulvar samples (Figure 6), *Lactobacillus* was the dominant genus at both time points in both lubricant groups, although its relative abundance was lower than typically observed in vaginal samples. Vulvar samples exhibited greater microbial diversity and higher abundance of anaerobic genera such as *Prevotella*, *Finegoldia*, and *Peptoniphilus*. Overall, changes in vulvar microbial composition over time were minimal and non-significant, further supporting the conclusion that lubricant use had little measurable effect on the vulvar bacteriome during the study period.

**Figure 7:**
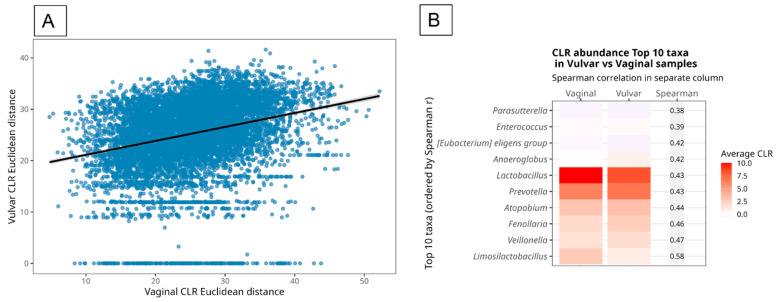
The Mantel test comparing Euclidean CLR distances between vaginal and vulvar bacteriome profiles shows a moderate but statistically significant correlation (Mantel *r* = 0.274, *p* = 0.001), indicating spatial specificity and yet association between the two sites. Each dot represents a pairwise comparison of samples and the trend line indicate a positive association between vaginal and vulvar bacteriome compositions (**A**). The heatmap shows the mean CLR abundance of the top 10 species with the highest correlation between vaginal and vulvar samples, sorted by ascending Spearman correlation coefficient (from top to bottom). Genera such as *Limnocylactobacillus*, *Villonella* and *Lactobacillus* showed strong positive correlations in abundance between sample types, supporting an overlap in community composition. Color intensity represents the average CLR abundance across samples and highlights species that are consistently shared across both locations (**B**).

Finally, Mantel’s test revealed a significant but modest correlation (*r* = 0.274, *p* = 0.001) between the vaginal and vulvar CLR Euclidean distance matrices. This correlation indicates a slight overlap in community structure (Figure 7A), implying that samples with a more diverse vaginal bacteriome also tend to have more diverse vulvar bacteriome. Heatmaps accompanied by Spearman correlations of CLR-transformed abundances showed that several genera, including *Limosilactobacillus*, *Veillonella*, *Fenollaria*, *Atopobium*, *Prevotella*, and *Lactobacillus*, were driving the significant correlation between anatomical sites (Figure 7B). Finally, correlation analyses with clinical variables (pH, BMI, and age) did not reveal significant associations, possibly due to the limited sample size included in this study, compared with previously published research [26].

## 4. Discussion

This study provides insight into the longitudinal effects of two personal lubricants, Tingling (water-based) and Warming (silicone-based), on the vaginal and vulvar bacteriome. The largely stable microbial communities observed over the intervention period suggest that short-term use of these products does not disrupt the ecological balance of these sites. The minor changes detected at the genus level suggest limited variation, but the overall vaginal and vulvar bacteriome remain resilient, reinforcing the idea that these products are unlikely to perturb established microbial ecosystems during 4 weeks of use.

The distinct patterns observed between vaginal and vulvar communities reflect the expected ecological differences between these anatomical sites [15,16,17,27]. Vaginal samples were predominantly *Lactobacillus*-dominated, consistent with a protective microbiome environment, while vulvar samples harbored a more diverse assemblage of genera including *Prevotella*, *Finegoldia*, *Peptoniphilus*, and *Corynebacterium*. These differences underscore the role of anatomical and physiological factors in shaping microbial communities and highlight the importance of considering site-specific microbiota when evaluating the impact of external products. Apparent non-significant increases in certain anaerobic genera in the Warming group are difficult to attribute directly to the lubricant composition, given baseline differences between lubricant groups and the compositional nature of relative abundance data, emphasizing that observed fluctuations may reflect natural variability rather than product effect.

Alpha and beta diversity patterns further support the resilience of these communities. Vulvar samples maintained higher diversity than vaginal samples, consistent with their exposure to skin and environmental microbes. Although a modest increase in vaginal diversity was noted in the Tingling group, these changes were not statistically significant, indicating that short-term use of the study lubricants did not meaningfully alter community structure. Beta diversity analyses confirmed that overall microbial composition remained stable across time points and sites. These findings suggest that short-term lubricant use does not compromise community integrity, and that the vaginal and vulvar microbiomes are resilient to transient environmental perturbations.

Comparisons with prior research contextualize these results. Previous studies have reported minimal impacts of water-based lubricants on vaginal microbiota, and differences between pre- and postmenopausal participants reflect baseline physiological variation rather than product-induced changes [19,26]. Case–control studies using self-collected swabs have similarly noted only subtle shifts in species-level abundance, often influenced by methodological factors such as sampling variability and lubricant heterogeneity [18]. Taken together, these observations reinforce the conclusion that the vaginal microbiome is resilient and that minor fluctuations in some taxa are likely influenced by host and environmental factors rather than lubricant formulation.

At the genus level, the consistent dominance of *Lactobacillus* across vaginal samples suggests that core protective taxa remain stable despite product use. Slight, non-significant shifts in anaerobic taxa and correlations between vulvar and vaginal communities highlight how less abundant taxa may exhibit subtle inter-site dynamics, but these do not compromise overall community stability. These findings reinforce the concept that both vaginal and vulvar microbiomes are governed more by intrinsic host factors than by the application of the two products tested in the present study [28].

The modest correlation observed between vaginal and vulvar microbiomes highlights the interconnectedness of microbial communities across adjacent anatomical sites. This relationship suggests that factors shaping the vaginal microbiome, such as host physiology and local microenvironment, may also influence the vulvar microbiome to some extent. The involvement of genera such as *Limosilactobacillus*, *Veillonella*, *Fenollaria*, *Atopobium*, *Prevotella*, and *Lactobacillus* showcases the mixed composition of the vulvar microbiome, which includes both mucosal- and skin-associated taxa [15,17,29]. These findings emphasize the importance of considering both anatomical sites when evaluating vulvovaginal health, as interactions between communities may contribute to resilience, stability, and overall microbial balance. Understanding these cross-site microbial relationships could inform future strategies for maintaining or restoring a healthy vulvovaginal microbiome, particularly in the context of interventions such as personal lubricant use.

Moreover, age and baseline microbial composition exert a stronger influence on bacteriome stability than lubricant use as revealed in a previous study [19]. The adaptability of the vaginal microbiome is primarily shaped by *Lactobacillus* populations and host regulatory mechanisms, including local pH, immunity, and the integrity of the mucosal barrier [28,30]. The minor, non-significant fluctuations observed in this study are consistent with the expected resilience of healthy vaginal and vulvar bacteriome and underscore the importance of considering both host and environmental contexts in microbiome research.

## 5. Strengths and Limitations

This study has several notable strengths. To our knowledge, this is the first study to characterize both vaginal and vulvar microbiome profiles from the same participants during the use of personal lubricants. The randomized, controlled design provides a robust framework for assessing the impact of personal lubricants on the vaginal and vulvar microbiome. The inclusion of participants across a broad age range, encompassing both premenopausal and postmenopausal women, enhances the generalizability of the findings throughout different stages of women’s life, as most prior studies on vaginal dryness have focused predominantly on postmenopausal populations. Sexual function was assessed using the FSFI questionnaire, a widely accepted and clinically validated instrument, ensuring reliable evaluation of participant-reported outcomes. The study also included novel assessments such as oral mucosal tolerance, as well as partner-reported measures via the subject perceived questionnaire and patient global impression of change, offering a comprehensive perspective on product effects all of which are available in the previous publication [21]. Nearly all participants successfully completed both the tolerance and treatment phases, with only one individual unable to finish, indicating high product tolerability and adherence. Additionally, participants were thoroughly trained by the Principal Investigator and authorized clinical site staff on proper application of the lubricants, which were provided on-site, supporting standardized use and minimizing procedural variability.

Several limitations of this study should be considered when interpreting the findings. The study population in each lubricant group (N = 33) was primarily composed of Caucasian participants, limiting racial and geographic diversity, which may influence microbiome profiling and reduce generalizability. A more diverse participant pool would have allowed for a more comprehensive assessment of baseline vaginal and vulvar bacteriome distributions across lubricant groups. The sample size, however, was determined to ensure adequate statistical power for the primary clinical endpoint (a prespecified increase in FSFI ≥4 points from baseline) [21]. All participants experienced mild-to-moderate vaginal dryness, which restricts the applicability of the results to the broader population without such symptoms. The relatively short 4-week duration limits the ability to assess potential long-term effects of lubricant use on the vaginal and vulvar bacteriome. Because participants were not restricted from condom use, potential influences of the partner’s penile microbiome on subtle vaginal or vulvar microbiome changes were not captured, representing an aspect that future studies should address. Finally, the use of 16S rRNA gene sequencing targeting only the V1–V3 region provided genus-level resolution but limited species-level discrimination and functional inference; employing a longer-read 16S approach covering the full V1–V9 region could yield deeper insights into species composition and potential functional shifts within the vulvovaginal bacteriome.

## 6. Conclusions

This study demonstrates that the vaginal bacteriome remained remarkably stable in composition, diversity, and community structure following four weeks of repeated exposure to either a water-based or silicone-based lubricant, with only modest and non-significant fluctuations observed in the vulvar bacteriome. The significant correlation between vaginal and vulvar communities further suggests coordinated microbial stability across adjacent genital sites. Collectively, these findings support the microbiome safety of well-formulated personal lubricants during regular sexual activity and provide evidence that such products are unlikely to disrupt the vulvovaginal microbial environment in the short term.

Given the importance of maintaining a healthy genital microbiome, these results may help inform product safety guidelines by reinforcing that lubricant formulations without disruptive excipients can be compatible with microbial stability. Nevertheless, larger, longer-term studies, including those incorporating metagenomic, functional, or mechanistic assessments, are needed to fully characterize potential subtle or cumulative effects and to ensure safety across diverse populations and usage patterns.

## Data Availability

The original data presented in the study are openly available in NCBI sequence archive under the BioProject accession number PRJNA1338715. Demographic information of the participants are available in the Supplementary Material of previously published study [21].

## Supplementary Materials

The following supporting information can be downloaded at https://www.mdpi.com/article/10.3390/microorganisms14010082/s1: Table S1: Summary of alpha variation differences between baseline and final time points for each lubricant group and anatomical site. Table S2: Summary of beta diversity analyses of variance (PERMANOVA and PERMDISP) comparing baseline and final time point for each lubricant group and anatomical region. Table S3: Linear mixed-effects (LME) models on centered log ratio (CLR) of vaginal samples pairwise baseline and final time points for each lubricant group. Table S4: Linear mixed-effects (LME) models on centered log ratio (CLR) of vulvar samples pairwise contrasts between baseline and final time points for each lubricant group. Table S5: Age associated microbiome taxa summary of vaginal samples using Linear mixed-effects (LME) models with central log ratio (CLR) transformed frequencies. Table S6: Age associated microbiome taxa summary of vulvar samples using Linear mixed-effects (LME) models with central log ratio (CLR) transformed frequencies. Table S7: Body mass index (BMI) associated microbiome taxa summary of vaginal samples using Linear mixed-effects (LME) models with central log ratio (CLR) transformed frequencies. Table S8: Body mass index (BMI) associated microbiome taxa summary of vulvar samples using Linear mixed-effects (LME) models with central log ratio (CLR) transformed frequencies. Table S9: pH associated microbiome taxa summary of vaginal samples using Linear mixed-effects (LME) models with central log ratio (CLR) transformed frequencies. Table S10: pH associated microbiome taxa summary of vaginal samples using Linear mixed-effects (LME) models with central log ratio (CLR) transformed frequencies. Table S11: Mean relative abundance of bacterial phyla in vaginal and vulvar samples.
